# Interpersonal predictive coding, not action perception, is impaired in autism

**DOI:** 10.1098/rstb.2015.0373

**Published:** 2016-05-05

**Authors:** T. von der Lühe, V. Manera, I. Barisic, C. Becchio, K. Vogeley, L. Schilbach

**Affiliations:** 1 Department of Psychiatry, University Hospital Cologne, 50937 Cologne, Germany; 2 CoBtek Laboratory, University of Nice Sophia Antipolis, 06103 Nice, France; 3 Cognitive Science Department, ETH Zürich, 8092 Zürich, Switzerland; 4 C'MON Cognition Motion and Neuroscience Unit, Fondazione Istituto Italiano di Tecnologia, Genova, Italy; 5 Department of Psychology, University of Turin, Turin, Italy; 6 Research Centre Juelich, Institute of Neuroscience and Medicine (INM-3), 52428 Juelich, Germany; 7 Max Planck Institute of Psychiatry, 80804 Munich, Germany

**Keywords:** predictive coding, high-functioning autism, social interaction, intention recognition

## Abstract

This study was conducted to examine interpersonal predictive coding in individuals with high-functioning autism (HFA). Healthy and HFA participants observed point-light displays of two agents (A and B) performing separate actions. In the ‘communicative’ condition, the action performed by agent B responded to a communicative gesture performed by agent A. In the ‘individual’ condition, agent A's communicative action was substituted by a non-communicative action. Using a simultaneous masking-detection task, we demonstrate that observing agent A's communicative gesture enhanced visual discrimination of agent B for healthy controls, but not for participants with HFA. These results were not explained by differences in attentional factors as measured via eye-tracking, or by differences in the recognition of the point-light actions employed. Our findings, therefore, suggest that individuals with HFA are impaired in the use of social information to predict others' actions and provide behavioural evidence that such deficits could be closely related to impairments of predictive coding.

## 1. Introduction

Action perception is not simply a reflection of what happens, but a projection of what will happen next. When we observe an action performed by another individual, our visual system anticipates how the action will unfold. Remarkably, body motion can represent a rich and reliable source of information [1]. Observers are capable of predicting the outcome of an observed action when viewing body movements even in the absence of contextual information. For instance, by looking at a point-light display of a person throwing a stone, observers can correctly judge the location targeted by the throw [2]. In more complex situations, movement observation can enable the observer to predict the other person's intentions. For example, from seeing a point-light display of someone grasping an object, observers can anticipate whether the object is grasped with the intent to cooperate, compete, or perform an individual action [3,4].

Recent evidence suggests that action perception based on body motion is crucial not only for interpreting the actions of individual agents, but also to predict how, in the context of an interaction between two agents, the actions of one agent relate to the actions of a second agent. In a seminal study, Neri *et al.* [5] demonstrated that when observing interactive activity requiring close body contact between two agents (such as fighting and dancing), the human visual system relies on the spatio-temporal coupling between two agents to retrieve information relating to each agent individually. Interestingly, the same holds true for social interactions that do not imply close body contact: observing the communicative gesture of one agent enhances the visual discrimination of a second agent responding to this communicative gesture, a phenomenon that has been referred to as ‘interpersonal predictive coding’ [4,6,7].

This study was designed to investigate interpersonal predictive coding in individuals with high-functioning autism (HFA). Individuals with autism show a reduced ability in reporting subjective and emotional states from point-light animations [8,9]. Whether they are also poor at understanding the actions of others from biological motion cues, however, is controversial. While some studies report impaired action recognition [10], other studies suggest that HFA observers do not differ from typical observers [9,11]. Similarly, while some studies report a lower sensitivity in detecting biological motion in individuals with HFA [12,13], others find no difference from control individuals' performance [14–16]. Cusack *et al.* [17] recently employed point-light stimuli of two interacting agents (fighting or dancing) to analyse the performance of individuals with HFA in a set of well-controlled tasks, spanning from low-level biological motion detection to action recognition and the ability to distinguish synchronized versus non-synchronized action sequences. They found no evidence of impairment in any of the tasks, thus, suggesting that persons with HFA are able to discriminate intact versus scrambled biological motion sequences, to discriminate one form of interaction from another, and even to discriminate between two agents who are acting in a synchronous way from those who are not.

However, clinical insight and marked impairments of social functioning in everyday life suggest that individuals with HFA fail to exploit such biological motion signals for the purposes of typical social interactions. Possibly because of this, some studies have suggested that individuals with autism spend less time attending to social cues compared with healthy controls (HCs) [18], and that autistic symptom severity may be related to reduced fixations of stimuli [19].

In this study, we used quantitative psychophysical measurements to investigate the modulatory effects of biological motion signals on perceiving a second agent while controlling for the role of low-level attentional factors through simultaneous eye-tracking. Our results show that despite being able to discriminate correctly between communicative and individual non-communicative action sequences when explicitly prompted to do so in a separate task, participants with HFA are not automatically using the action of one agent as a predictor of the action of an interacting partner who does not stand in physical contact with the first.

## 2. Material and methods

### (a) Participants

In order to determine the sample size of our study to detect an interaction between group (HFA versus HC) and condition (communicative (COM) versus individual (IND)) in the interpersonal detection task (*d′*), we performed a power analysis. Assuming a medium effect size (partial eta-squared = 0.06), and a correlation between repeated measures = 0.80 (obtained in a pilot study), we set power at 0.95 to avoid a possible type II error. Correlation between repeated measures for the interpersonal detection task was estimated in a pretest run of 14 healthy participants (nine females and five males; age: *M* = 27.4, s.d. = 1.9; education: *M* = 17.1, s.d. = 0.9). Repeated-measures ANOVA on *d′* with condition (COM versus IND) as a within-subject factor revealed a significant main effect of condition (*F*_1,13_ = 6.61, *p* = 0.023, partial *η*^2^ = 0.34), with participants showing a higher discrimination performance in the COM condition (*M* = 1.55, s.d. = 0.83) compared with the IND condition (*M* = 1.21, s.d. = 0.67). A significant correlation between *d′* in the COM and IND condition was found (*r*_13_ = 0.80, *p* = 0.001). The power analysis conducted in G*Power [20,21] determined that a total sample of 32 participants was needed to obtain power = 0.95, *α* = 0.05, two-tailed. Consequently, 16 adults with HFA and 16 HCs were recruited for this study. The two groups were closely matched for age, sex, years of education, and IQ as measured by WST (*Wortschatztest,* German multiple-choice vocabulary test, [18]). The group of HC reported no history of neurological or psychiatric disorders and no current use of psychoactive medications. Furthermore, they were only included if they had an autism spectrum quotient (AQ) below 23 [22] and a Beck depression inventory (BDI) score of 17 or below [23]. All HFA participants were diagnosed and recruited in the Autism Outpatient Clinic at the Department of Psychiatry and Psychotherapy, University Hospital of Cologne in Germany. Clinical consensus diagnosis was established using the international classification of diseases (ICD-10) criteria by two clinicians specialized in autism diagnosis in adulthood, who explored each individual patient in an independent interview and examination. All diagnoses were confirmed by one of two senior psychiatrists specialized in autism. Patients with a diagnosis of childhood autism (F84.0) and Asperger syndrome (F84.5) were included when average or above-average IQ had been ascertained. As depression is a common co-morbidity in HFA [24,25], autistic participants with a BDI score above 17 or a history of depression were not excluded from the study although this resulted in a significant difference in the BDI scores between HFA and HC. To control for depression symptoms, correlations with BDI scores were included during data analysis (for details, see the Data Analysis section). In accordance with the clinical diagnosis, there were significant differences in the autism spectrum quotient [26] between HFA and HC (table 1).

**Table 1.** Demographic and neuropsychological variables of control and patient group. IQ was assessed by a German multiple-choice vocabulary test (*Wortschatztest*, WST) [27], which allows for a quick and valid estimation of general intelligence [28,29]. s.d., standard deviation.

	HFA *n*=16	HC *n*=16	*t*-test
sex ratio (female : male)	4 : 12	6 : 10	*t*_30_ = 0.75, *p* = 0.46
mean age (s.d.)	41.56 (9.15)	36.19 (12.11)	*t*_28_ = 1.42, *p* = 0.17
mean years in education (s.d.)	18.63 (4.91)	18.94 (2.72)	*t*_23_ = −0.22, *p* = 0.83
mean IQ (s.d.)	116.88 (15.59)	115.31 (8.43)	*t*_23_ = 0.35, *p* = 0.73
mean BDI (s.d.)	16.44 (11.45)	3.88 (4.56)	*t*_30_ = 4.08, *p* < 0.01
mean AQ (s.d.)	40.50 (5.83)	14.19 (6.91)	*t*_30_ = 11.64, *p* < 0.01

### (b) Interpersonal detection task

#### (i) Stimuli

**Figure 1.**
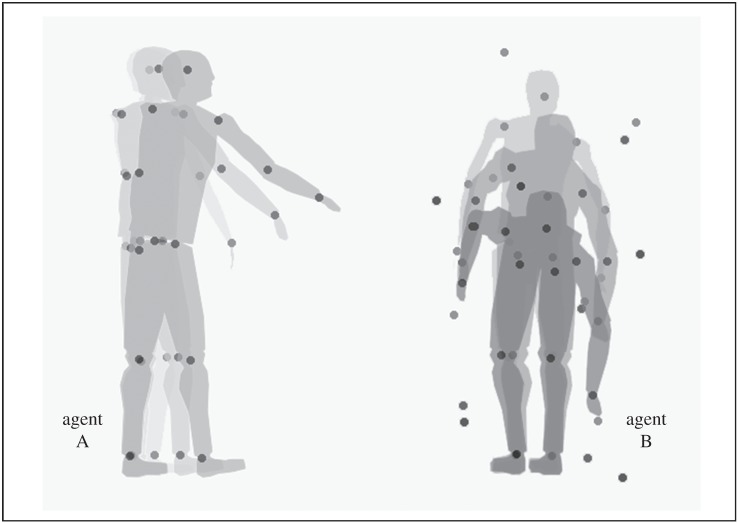
Example of a communicative signal trial. Agent A points to an object to be picked up; agent B bends down and picks it up. Agent B was presented using limited-lifetime technique (six signal dots) and masked with temporally scrambled noise dots. The noise level displayed is the minimum allowed in the experiment (five noise dots). To provide a static depiction of the animated sequence, dots extracted from three different frames are superimposed and simultaneously represented; the silhouette depicting the human form was not visible in the stimulus display. (Adapted from [6]).

Stimuli consisted of two point-light walkers, each made up of 13 markers indicating the major joints of the actor. These stimuli were selected from the communicative interaction database—5AFC format (CID, [30,31]). Six point-light stimuli were employed, three belonging to the COM condition (‘squat down’, ‘look at the ceiling’, and ‘sit down’) and three belonging to the IND condition (‘turn over’, ‘sneeze’, and ‘drink’). COM stimuli showed a communicative interaction between two agents, with an agent (A) performing a communicative gesture towards a second agent (B), who responded accordingly (figure 1). Stimuli for the IND condition were created by substituting agent A's communicative action with a non-communicative action with the same onset and duration.

#### (ii) Apparatus

Eye movements were recorded using a Tobii T60 eye-tracker with a sampling frequency of 60 Hz. Stimuli were presented on the integrated 17-inch TFT monitor with resolution set to 1280 × 1024 pixels. A five-point eye calibration was run before the beginning of each of two blocks in the main experiment. Participants were tested individually in a dimly lit and sound-attenuated room. Participants were seated at a viewing distance of 60 cm from the screen, and were asked to sit as still as possible. However, they were not restrained in their head and trunk movements.

#### (iii) Training session

Before the detection task, the number of noise dots was adjusted individually for each participant during a training session. Stimuli consisted of three actions selected from the CID, masked with five levels of noise (zero, five, 10, 20, or 40 noise dots). The actions were different from those used in the main experiment. Each participant completed two blocks of 60 trials each (four repetitions of three actions by five noise levels). After completing the second block, individual noise levels were determined by fitting a cumulative Gaussian function to the proportion of correct responses and determining the 70% threshold. The minimum noise level allowed was five noise dots.

#### (iv) Experimental procedure

*Interpersonal detection task.* A two-alternative forced-choice (2AFC) paradigm was employed: each trial consisted of two intervals, a ‘target’ interval (containing agent B) and a ‘non-target’ interval (not containing agent B), separated by the presentation of a 500 ms fixation cross. Depending on the action stimulus, the duration of each interval ranged from 3600 to 4333 ms (*M* = 3978 ms, s.d. = 0.367 ms). In the target interval, B's actions were displayed using a limited-lifetime technique and masked with limited-lifetime noise dots [5,32]. This technique was used to prevent observers from using local motion or position cues to perform the task [33]. Each signal dot was presented for 200 ms at one of the 13 possible locations, then disappeared and reappeared at another randomly chosen location. Only six signal dots per frame were shown simultaneously. Dot appearance and disappearance were asynchronous across frames. Noise dots had the same trajectories, size, and duration as the signal dots, but were temporally and spatially scrambled. The number of noise dots was adjusted individually for each participant during a pretest session (for further details, see ‘Training session’).

**Figure 2.**
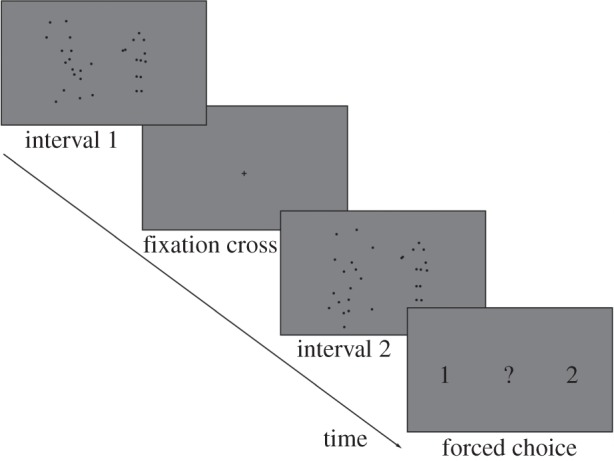
Schematic of trial structure. After seeing the stimuli during two intervals (interval 1 and 2)—separated by the presentation of a fixation cross (500 ms)—participants were asked to decide which interval contained agent B. The maximum response time was 2000 ms.

In the non-target interval, agent B was substituted by a scrambled version of the corresponding signal action obtained by temporally scrambling the relevant dots. Noise dots were also added in order to obtain the same number of dots as displayed in the signal interval. On average, positions and motions of the dots in the non-target interval equalled those of the target interval [5]. In both the target and the non-target intervals, agent A was neither masked nor limited-lifetime (figure 2).

After seeing the two intervals during each trial, participants were asked to decide which interval contained agent B. Responses were given by pressing one of two marked keys on a keyboard (maximum response time = 2 s). In line with previous results, we hypothesized that the ability to detect agent B would be enhanced in the communicative condition. This is because this condition includes complementary actions, which means that the action of agent A can be used to predict the action of agent B. Each participant completed two blocks of 96 trials each (16 repetitions of three actions in two conditions). Both blocks comprised trials of both conditions presented in a randomized order. Blocks lasted approximately 15 min each and were separated by a rest period of 3 min. After completion of each block, participants were informed about their current percentage of correct responses.

*Recognition task.* After completion of the detection task, participants were administered an explicit intention recognition task. Stimuli consisted of 21 videos of point-light actions depicting two point-light agents (neither limited-lifetime nor masked) selected from the communicative-interaction database—5AFC format (31), including 14 communicative actions (COM: ‘come closer’, ‘squat down’, ‘walk away’, ‘imitate me’, ‘look at the ceiling’, ‘look at the floor’, ‘go out of the way’, ‘no’, ‘pick this up’, ‘move this down’, ‘sit down’, ‘stand up’, ‘stop’, and ‘choose which one’) and seven individual actions (IND: ‘turn over’, ‘jump’, ‘sneeze’, ‘lateral steps', ‘drink’, ‘stretch’, and ‘look under the foot’). The six actions (three COM, three IND) used in the detection task were included in the list. Stimuli were presented in a randomized order. Every video was presented twice consecutively. After the second repetition of each video, participants were asked three questions: (i) to report whether the action had been presented in the interpersonal detection task; (ii) to decide whether the two agents were communicating versus acting independently of each other; and (iii) to select the correct action description among five response alternatives, presented in German [31]. The five alternatives were assembled by replacing the correct description of agent A's action (e.g. A asks B to walk away) with two incorrect communicative alternatives (e.g. A opens the door for B; A asks B to move something) and two incorrect non-communicative alternatives (A stretches; A draws a line). Questions were presented on the screen until response, with no time restriction. No feedback concerning response correctness was given to the participants.

#### (v) Data analysis

The behavioural measurements obtained during the experiment were recorded and later analysed by using MATLAB scripts (MathWorks, Natick, MA) and Predictive Analytics software v. 18 (PASW18; www.spss.com).

*Interpersonal detection task.* For each participant, we calculated the proportion of hits (defined as ‘second interval’ responses when the target was in the second interval) and false alarms (second interval responses when the target was in the first interval) in the two experimental conditions to estimate the signal detection theory (SDT) parameters sensitivity (*d′*) and criterion (*c*) in the two experimental conditions [34]. Sensitivity is a measure of the individual's ability to discriminate whether the signal (here, agent B) is presented in the first or in the second interval. Higher values of *d′* (ranging from 0 to + ∞′) indicate better discrimination ability. The response criterion *c*, also known as ‘response bias’, reflects the tendency to report that the signal (here, agent B) is presented in the first or the second interval. In 2AFC tasks, the criterion does not usually differ from zero, which indicates no systematic tendency to respond ‘first interval’ or ‘second interval’. Proportions of 0 were replaced with 0.5/*N*, and proportions of 1 were replaced with (*N* − 0.5)/*N* (where *N* is the number of first interval and second interval trials).

Paired sample *t*-tests were used to investigate condition-specific differences in sensitivity and criterion for each group separately. To evaluate condition- and group-specific differences in sensitivity and criterion as well as their statistical interaction, we used a mixed repeated-measure ANOVA employing the within-subject factor ‘condition’ (COM versus IND) and a between-subject independent variable ‘group’ (HFA versus HC). In order to rule out a possible influence of BDI scores on task performance, the BDI scores were used for correlation analyses with sensitivity measurements from all experimental conditions. Furthermore, AQ scores were used for correlation analyses with sensitivity measurements to estimate the relationship between the degree of ‘autistic traits’ and task performance.

Python software (Python Software Foundation, v. 2.7.3150) was used to extract and post-process gaze position and pupil size data over time, as measures of visual attention and arousal, respectively. Owing to technical problems and excessive head movements, data from 12 HFA participants and 13 HC participants could be included in the gaze data analysis. To ensure that all participants were engaged in the task and attended both presented agents (A and B), we extracted the number and position data of gaze events in two regions of interest (ROI): a right ROI where agent A was located and a left ROI where agent B was located. The ROIs were defined by fitting the smallest possible rectangle onto the visual display that comprised all stimulus dots on each side of the stimulus screen. In order to explore potential differences in eye movements between groups and conditions, the number of fixations as well as the number of gaze shifts between right ROI and left ROI was calculated. To detect fixations, a dispersion-threshold algorithm was used [35], with a dispersion threshold of 43 pixels and a minimal fixation duration of 100 ms. Gaze shifts were defined as two sequential fixations falling onto different ROIs. Measures of pupil size were directly provided by the Tobii T60 system and also recorded during the entire duration of the main test.

*Recognition task.* In order to assess whether participants were able to recognize COM and IND actions when these were not masked by noise dots—including those six actions which had been part of the detection task—we computed the percentage of correct responses for each of the three questions, and we compared the mean performance across the two groups by means of independent sample *t*-tests.

## 3. Results

### (a) Interpersonal detection task

The mean proportion of correct responses in the main experiment was 0.64 (s.d. = 10.74) in the HC group and 0.69 in the HFA group (s.d. = 9.86). This indicates that the number of noise dots selected in the training session was sufficiently accurate for the participants of both groups. No significant difference between the mean number of noise dots was found between the HFA group (*M* = 10.56, s.d. = 9.32) and the HC group (*M* = 14.63, s.d. = 9.32) (*t*_30_ = −1.23; *p* = 0.23, partial *η*^2^ = 0.05). Similarly, no difference in the criterion (*c*) parameter was found between the HFA group (*M* = 0.028; s.d. = 0.26) and the HC group (*M* = −0.13; s.d. = 0.28) (*t*_30_ = 1.61; *p* = 0.12, partial *η*^2^ = 0.08).

**Figure 3.**
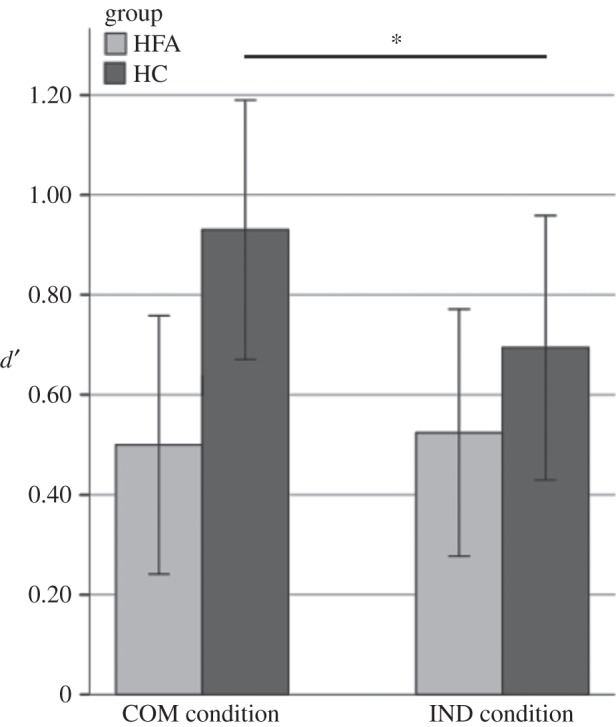
Mean sensitivity (*d′*) across groups and conditions. Error bars depict 95% confidence intervals.

Repeated-measures ANOVA revealed a significant main effect of condition (*F*_1,30_ = 4.93, *p* = 0.034, partial *η*^2^ = 0.14), with higher sensitivity in the COM (*M* = 0.72, s.d. = 0.53) than in the IND condition (*M* = 0.61, s.d. = 0.48). However, this was moderated by a significant interaction effect between condition and group (*F*_1,30_ = 7.45, *p* = 0.011, partial *η*^2^ = 0.20). To break down this interaction, a simple effects analysis was performed, which demonstrated a significant effect of condition in the HC group (*F*_1,15_ = 12.25, *p* = 0.001, partial *η*^2^ = 0.29), whereas no such effect was observed in the HFA group (*F*_1,15_ = 0.13, *p* = 0.721, partial *η*^2^ = 0.004; figure 3). In the light of a smaller number of participants for whom eye-tracking data were available, we repeated our repeated-measures ANOVA in this sample (12 patients, 13 controls) and also found the significant interaction effect between condition and group (*F*_1,23_ = 5.14, *p* = 0.033, partial *η*^2^ = 0.183).

To further corroborate the hypothesis of an interaction between group and condition, we conducted a Bayes factors analysis [36,37]: applying the JZS Bayes factor method suggested by Rouder and co-workers [36] to our sensitivity data (with default scale factor 1.0) yields a Bayes factor of 4.7, meaning that the hypothesis of an interaction between group and condition is almost five times more probable than the null hypothesis of an absence of interaction. As a factor in excess of 3.2 is conventionally considered to provide ‘substantial’ evidence in favour of a hypothesis [37], the Bayesian factor analysis suggests that the present results are unlikely to be due to a type II error.

**Figure 4.**
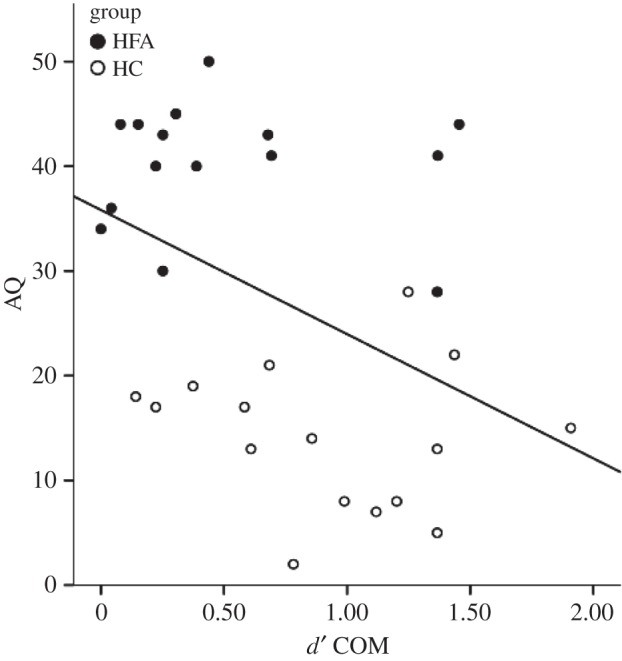
Correlation between AQ and *d′* in the COM condition across groups.

Correlation analyses of BDI scores with measures of sensitivity across all experimental conditions and groups did not show any significant results (maximum *r* = −0.25, minimum *p* = 0.17). Correlation analyses of AQ with measures of sensitivity across both groups did show a significant negative correlation between AQ and *d*′ in the COM condition (*r* = −0.422, *p* = 0.016; figure 4).

Eye-tracking data showed that both HC and HFA visually inspected both agents. Consistently, we found no significant between-group difference for either total number of fixations (HFA: *M* = 1250.83, s.d. = 503.33; HC: *M* = 898.92, s.d. = 454.59, *t*_23_ = 1.84, *p* = 0.79) or gaze shifts from one ROI to the other (HFA: *M* = 449.17, s.d. = 354.22; HC: *M* = 317.31, s.d. = 136.73, *t*_23_ = 1.25, *p* = 0.23). No between-group difference was observed for pupil size across all experimental conditions (HFA: *M* = 2.98, s.d. = 0.28; HC: *M* = 3.14, s.d. = 0.52, *t*_17_ = −0.97, *p* = 0.34). In order to compare the number of fixations across the two ROIs for both groups, we performed a mixed repeated-measures ANOVA, which demonstrated a significant main effect of ROI (*F*_1,23_ = 20.75, *p* < 0.001) with more fixations in the left ROI containing agent B (*M* = 789.32, s.d. = 425.99) than in the right ROI containing agent A (*M* = 278.52, s.d. = 307.62). There was no significant interaction effect between fixations in each ROI and group (*F*_1,23_ = 3.38, *p* = 0.079). No significant difference between the COM and the IND condition was found in pupil size or in gaze behaviour across groups.

### (b) Recognition task

*Question* (*1*): no significant difference was found between HFA and HC participants in the ability to correctly identify which COM and IND actions had been presented in the previous detection task (mean proportion of correct responses in HFA: *M* = 0.62, s.d. = 0.36; HC: *M* = 0.80, s.d. = 0.20, *t*_30_ = −1.82; *p* = 0.08).

*Question* (*2*): no difference between groups was found in the ability to classify the stimuli employed in the detection task (*n* = 6) as communicative versus non-communicative (HFA: *M* = 0.88, s.d. = 0.13, HC: *M* = 0.87, s.d. = 0.13, *t*_30_ = 0.23, *p* = 0.82). The same was true for the actions not presented in the detection task (*n* = 15) (HFA: 0.89, s.d. = 0.07, HC: 0.91, s.d. = 0.08), *t*_30_ = −0.67, *p* = 0.51).

**Figure 5.**
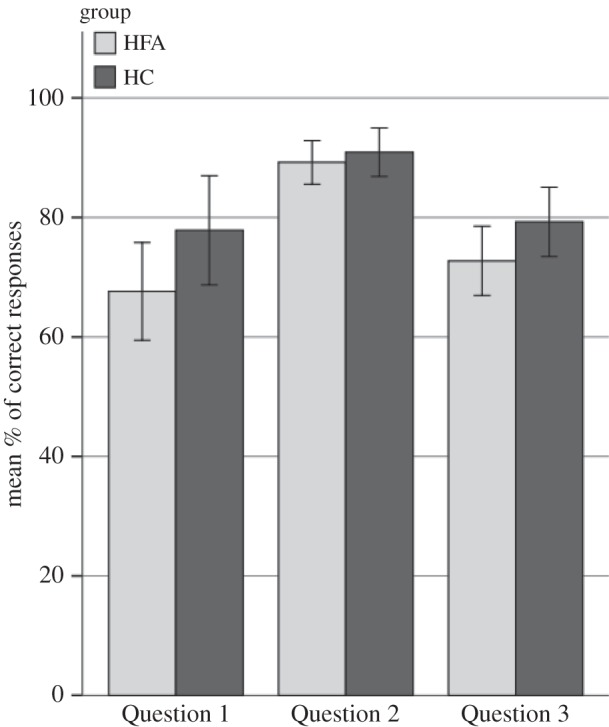
Bar graphs of correct responses during post-test questionnaire across groups. Error bars depict 95% confidence intervals. Question 1: ‘Did you see this action in the previous task?’ Question 2: ‘Are the two agents communicating or acting independently from one another?’ Question 3: ‘Which alternative best describes this action?’.

*Question* (*3*): no difference between HFA group and HC group was found in selecting which of the five response alternatives best described the observed actions (HFA: *M* = 0.73, s.d. = 0.11, HC: *M* = 0.79, s.d. = 0.11, *t*_30_ = −1.70, *p* = 0.10; figure 5).

## 4. Discussion

Previous studies have shown that in the context of interactive activities between two agents, the actions of one agent can be used as predictors of the actions of a second agent, which has been referred to as ‘interpersonal predictive coding’ (4). Corroborating these previous findings, healthy participants in the study described here made use of the communicative actions of a first agent (A) to predict the actions of a second agent (B). Critically, this form of interpersonal predictive coding was not found in a matched group of individuals with HFA. Autistic participants did not show any improvement in the communicative condition compared with the individual condition, in which A and B acted independently of each other. This finding stands in contrast to recent evidence suggesting that the HFA ‘perceptual system returns functionally intact signals for interpreting other people's actions adequately’ [17].

Importantly, our study allows a number of potentially confounding factors to be ruled out: first, because participants with autism in our study looked at agent A and B for the same amount of time as HCs in both the communicative and the individual condition, we can rule out the possibility that the difference in sensitivity across groups is due to differences in gaze behaviour. Second, performance on a second recognition task in our study excludes the possibility that lower performance in the detection task is explained by a general failure to recognize communicative intentions portrayed by point-light displays. When presented with the same stimuli employed in the detection task and asked to label them, autistic participants performed as well as HCs in distinguishing communicative and individual actions, and in selecting the correct action description. Third, correlational analyses of depressive symptoms as measured by the BDI and detection performance allows us to rule out an influence of co-morbid psychopathology on interaction perception.

The impairment of interpersonal predictive coding in individuals with autism was most likely owing to an inability to predict agent B's response based on agent A's communicative intention, whereas the ability to explicitly recognize agent A's communicative intentions was found to be intact. These findings are also consistent with recent evidence from computational modelling, which demonstrates that autistic traits in HCs are not related to a general inability to process social stimuli; rather, they are closely related to an inability to take advantage of social information during decision-making [38]. In line with these findings that span the entire spectrum of autistic traits, our study further demonstrates that the degree of autistic traits as measured by the AQ score [22] was negatively correlated with detection performance across *both* groups, such that participants with higher autistic traits showed decreased interpersonal predictive coding.

### (a) Interpersonal predictive coding and online social cognition

The finding that HFA participants show an impairment of interpersonal predictive coding has important implications for the understanding of online social cognition in autism [39,40]. When we are engaged in a direct social interaction with a partner, prediction of the other person's actions helps us adjust our movements ‘online’, i.e. in real-time, in order to plan an appropriate response and coordinate with her while observing her movements [41]. Such an inability to automatically integrate social information and use it to predict subsequent actions of conspecifics has been related to a potential deficit of predictive coding in autism [42]. This could be due to prior expectations that are built up through participation in social interactions and which help us to be responsive to others [40]. Accordingly, autistic observers might be unable to automatically situate a person in the context of forthcoming states and subsequent responses of a social interactor or respondent. This deficit of interpersonal action prediction and a resulting lack of social responsiveness may help to explain the discrepancy between intact social reasoning and recognition skills, and deficits in online social interaction in HFA [43]. On the other hand, and in the light of evidence demonstrating that expertise plays an important role in making accurate predictions when observing human actions [44], one could argue that extensive practice in social interaction may lead to more accurate predictions when observing communicative interaction dyads.

### (b) Interpersonal predictive coding in the brain

A growing body of experimental and theoretical work provides evidence that predictive coding is a neurobiologically plausible scheme [45], according to which different neural systems generate statistical predictions about the current state of our environment and then adjust them to the evidence ‘at hand’. In other words, ‘expectations have a strong and general influence on our experience of the sensory input’ [46]. Interestingly, under certain conditions prior expectations may be favoured over available sensory input [47]. Consistent with this, it has been demonstrated that prior expectations can have an effect on the processing of others' perceived actions that may be so strong as to generate the illusion of seeing an agent when no such agent is actually present, which has been referred to as seeing a ‘Bayesian ghost’ [6].

A Bayesian account of the so-called mirror neuron system of the brain suggests that an internal model is generated during action observation, which transmits an action prediction to representations in the superior temporal sulcus (STS) and parietal brain areas [48]. In the same line, a recent study provides evidence that within a predictable context, mirror neurons can discharge before the onset of an observed action [49]. Furthermore, recent research provides evidence that medial prefrontal cortex (mPFC) plays a key role in the top-down control of social signal processing (i.e. social top-down response modulation STORM [50,51]). Consistently, it was demonstrated that mPFC activity modulates brain activity in other regions, which are relevant for action control in a social context, such as inferior frontal gyrus [50,52]. Similarly, mPFC might also be involved in modulating brain activity relevant for the sensory processing of social stimuli in the STS. This modulation might convert an adaption of priors relevant for Bayesian inference, and could help explain the emergence of social perception in the absence of social stimuli. In the light of evidence demonstrating that the mPFC shows reduced activations in subjects with autism when they are processing social stimuli such as ‘social gaze’ [53] or evaluating the animacy of moving objects [52], the deficits in interpersonal predictive coding in autism described here might be due to an underlying abnormality in mPFC. Furthermore, it is conceivable that differences in long-range connectivity in autism may prevent mPFC-based modulations of temporoparietal regions relevant for the processing of biological motion. Future brain imaging studies could help to provide new insights into these modulatory processes by making use of the experimental paradigm described here.
